# Supplementary material for: Biomonitorization of concentrations of 28 elements in serum and urine among workers exposed to indium compounds
Source: PLoS One. 2021 Feb 22;16(2):e0246943. doi: 10.1371/journal.pone.0246943 (PMC7899351; doi:10.1371/journal.pone.0246943)
Supplement: S1 Questionnaire — (DOC) [file pone.0246943.s002.doc]

**NO.________**

**Occupational exposure questionnaire**

**General information**

Name: __________ Sex：（male female） Nation: ______________

Height：________cm Weight: kg Birth: ______________

Company：______________________ Length of service：______________

Address：________________________ Telephone: ______________

**Occupation history**

| factory | workshop | type of work in production | Start date | End date | Job description | concentration (mg/m^3^) | |
| --- | --- | --- | --- | --- | --- | --- | --- |
|  |  |  |  |  |  | first time | second time |
| 1. |  |  |  |  |  |  |  |
|  |  |  |  |  |  |  |  |
| 2. |  |  |  |  |  |  |  |
|  |  |  |  |  |  |  |  |

**Exposure to radiation**

| **X-ray fluoroscopy or radiography** | **time** | **Body parts** |
| --- | --- | --- |
|  |  |  |
|  |  |  |
|  |  |  |

**Past health / disease history：**____________________________________________

Commonly used drugs:_________________________________________________

Types of current medication： _________________________________________________

**Does the immediate family member have cancer：**(Yes No)

If so, what is the tumor ________________________

The relationship between the patient and you _________

**Smoking Status**

Smoking: Yes No

amount of cigarette smoking: 1-5 cigarettes / day 5-10 cigarettes / day

10-20 cigarettes / day > 20 cigarettes / day

Smoking type: filter cigarette Filter free cigarettes cigar < kg / month>

Age to start smoking_______ year

Total smoking _____ year

Quit smoking in______ year

Spouse smoking: Yes No

**Drinking situation**

Liquor drinking: no drinking, < 12 Liang / day, 1-5 Liang / day, > 5 Liang / day

Beer drinking: no drinking, < half bottle / day, 1-2 bottles / day, > 2 bottles / day

Drinking fruit wine: less than once, 2-4, 5-10, and more than 10 times

(Note: it is enough to investigate the situation within one year.)

**Dietary habit**

Vegetables 1. Rarely eat 2. Generally 3. Eat more 4. Eat a lot

Fruit 1. Rarely eat 2. Generally 3. Eat more 4. Eat a lot

Sauerkraut 1. Rarely eat 2. Generally 3. Eat more 4. Eat a lot

Fried food 1. Rarely eat 2. Generally 3. Eat more 4. Eat a lot

Carbon baked food 1. Rarely eat 2. Generally 3. Eat more 4. Eat a lot

Whether to supplement vitamin 1. Yes the name_____ 2 No

**Tea drinking**

Tea consumption: 1. Drink every day; 2. Drink often; 3. Drink occasionally; 4. Never drink tea

Types of tea: 1. Green tea 2. Black tea 3. Scented tea

Tea together___________ year

**Chemicals exposed to the working area:**

1. Dust 2. Lead 3. Hydrochloric acid 4. Arsenic 5. Cadmium 6. Sodium hydroxide 7. Zinc 8. Indium 9. Hydrogen arsenide 10. Indium sulfate 11. Indium oxide 12. Indium chloride

13. Others:____________________________________________

**Do you often contact with other heavy metals such as**

1. Aluminum 2. Cadmium 3. Chromium 4. Manganese 5. Nickel

6. Others:_____________________________________________

Name of investigator ___________ Date of investigation ______________

**编号：________**

**职业接触调查表**

**一般情况**

姓名: __________ 性别：（男 女） 民族: ______________

身高：__________cm 体重: kg 出生: ______________

单位：__________________________________ 工龄：______________

住址：__________________________________ 电话: ______________

职业史

| 工厂名称 | 车间 | 工种 | 开始年月 | 结束年月 | 任务描述 | 浓度(mg/m^3^) | |
| --- | --- | --- | --- | --- | --- | --- | --- |
|  |  |  |  |  |  | 第1次 | 第2次 |
| 1. |  |  |  |  |  |  |  |
|  |  |  |  |  |  |  |  |
| 2. |  |  |  |  |  |  |  |
|  |  |  |  |  |  |  |  |

接触放射线情况

| **X射线透视或拍片** | **时间** | **身体部位** |
| --- | --- | --- |
|  |  |  |
|  |  |  |
|  |  |  |

**既往健康/疾病史**：_________________________________________________

常用的药物: _________________________________________________

现服用药物种类： _________________________________________________

**直系亲属是否患有肿瘤**：(是 否) 如有是何肿瘤________________________

患者与你的关系_________

**吸烟情况**

是否吸烟: 是 否

吸烟量: 1-5支/天 5-10支/天 10—20支/天 >20支/天

吸烟种类: 过滤嘴香烟 无过滤嘴香烟 卷烟 < 斤/月>

开始吸烟年龄_______(周岁) 共吸烟_____年 已戒烟______年

配偶是否吸烟: 是 否

**饮酒情况**

饮白酒情况:不饮、 <1两/天、 1—5两/天、 >5两/天

饮啤酒情况:不饮、 <半瓶/天、 1—2瓶/天、 >2瓶/天

饮果酒情况:少于1次、 2—4次、 5—10次、 10次以上

（注：调查一年内的情况即可）

**饮食习惯**

蔬菜 1.平时极少吃 2.一般 3.吃得较多 4.吃得很多

水果 1.平时极少吃 2.一般 3.吃得较多 4.吃得很多

酸菜 1.平时极少吃 2.一般 3.吃得较多 4.吃得很多

油炸食品 1.平时极少吃 2.一般 3.吃得较多 4.吃得很多

碳烤食品 1.平时极少吃 2.一般 3.吃得较多 4.吃得很多

**平时是否补充维生素** 1.是 名称_____ 2 否

**喝茶情况**

喝茶量: 1.天天喝 2.经常喝 3.偶尔喝 4.从不喝茶

经常喝茶的种类: 1.绿茶 2.红茶 3.花茶

共喝茶___________年

**工作区域接触的化学物:**

1. 粉尘 2. 铅 3.盐酸 4. 砷 5. 镉 6.氢氧化钠 7. 锌

8. 铟 9. 砷化氢 10.硫酸铟 11.氧化铟 12.氯化铟

13.其他: ____________________________________________

除工作区域外是否经常接触其它重金属如

1.铝 2.镉 3.铬 4.锰 5.镍

6.其他: _____________________________________________

调查员姓名 ___________ 调查日期 ______________
