# Supplementary material for: Biomonitorization of concentrations of 28 elements in serum and urine among workers exposed to indium compounds
Source: PLoS One. 2021 Feb 22;16(2):e0246943. doi: 10.1371/journal.pone.0246943 (PMC7899351; doi:10.1371/journal.pone.0246943)
Supplement: Attachment — Submitted filename: Reviewer_Comment.pdf [file pone.0246943.s005.pdf]

[Overall comment]

Thank you for the second revision of the manuscript. More revisions have been made in more places and we believe that the research will be more qualified to the reader. However, we still have concerns about some of the comments. Also, there are some points that should be pointed out in the revised manuscript. We ask that you please review and revise the manuscript.

It would also help me to complete this review more quickly if the rebuttal letter could be more specific as to which parts of the manuscript have been changed.

For the sake of brevity, I have used the following abbreviations in this review comment.

- Manuscript (Ori) = Original manuscript
- Manuscript (R1) = Manuscript (1st revised version)
- Manuscript (R2) = Manuscript (2nd revised version)
- Rev-com (Ori) = My Reviewer comment for manuscript (Ori)
- Rev-com (R1) = My reviewer comment for manuscript (R1)
- Rev-com (R2) = This reviewer comment
- Rebuttal letter (R1) = Rebuttal letter attached manuscript (R1), i.e. response to Rev-com (Ori)
- Rebuttal letter (R2) = Rebuttal letter attached manuscript (R2), i.e. response to Rev-com (R1)

To avoid any misunderstandings, the following definitions of the terms should also be stated

- Unexposed workers = Control subjects
- Study subjects = indium-exposed workers and unexposed workers selected for this study.

[Ongoing concerns]

3-r2. Multiple statistical testing Problem (False positive rate will be extremely high)

The problem is that a False positive rate will be extremely high.

As the authors responded to in their rebuttal letter (R2), small sample size is one of the reasons why we cannot control for random errors. Manuscript (R2) pointed out this.

However, the small sample size is not the root cause of the problem of false positives. The authors should take into account the high number of statistical tests. Even if one

increases the sample size, if it is not infinite, the same problem arises if the number of tests is high.

This study is an exploratory study, not a hypothesis-driven study. Adjustment of p-values (and confidence intervals) is usually unrealistic when large numbers of statistical tests are performed in an exploratory analysis. In such situations, it is difficult to accurately quantify the total number of tests performed and their interconnectedness, and the adjusted threshold for statistical significance is very small (and the false-negative rate is very high) because of the large number of tests performed. Therefore, in an exploratory analysis, it is important to interpret results cautiously rather than trying to accurately determine the true significance level of the p-value.

My two main concerns in the analysis of this study are ;

- Problem A) No statistical adjustment for p-values has been made.
- Problem B) There is no careful interpretation of the p-values.

In Rev-com (R1) comment No *3-r1, I commented that this is a way to deal with Problem A) and B)

- Please address it statistically.
- Please consider this issue in the Discussion section.

I consider this study to be an exploratory study. However, since neither method was selected in Manuscript(R2), this falsely positive issue remains. As shown above, it is very difficult to adjust the p-value in exploratory studies, so please be careful in interpretation of the p-value and clearly state it as a Limitation in the Discussion section. Also, in the Method section, you should state that this is an exploratory study.

Also, if this study was not conducted as a exploratory study, please adjust the p-value.

There are many papers on this issue. Please refer to the following and others.

- Schochet, P. Z. (2009). An approach for addressing the multiple testing problem in social policy impact evaluations. *Evaluation Review*, 33(6), 539-567.
- Sainani, K. L. (2009). The problem of multiple testing. *PM&R*, *1*(12), 1098-1103.

*13-r2. Are the study subjects exposed to any other toxic metals?

This is a point that was mentioned in Rev-com (Ori) comment No 13 and responded to in a rebuttal letter (R1). The response stated "the indium-exposed workers were exposed to other toxic metals, too." in the response; also in Manuscript (R2), in the

introduction, the authors stated that " In the process of indium smelting, there are occupational hazards such as dust, lead, arsenic, cadmium, indium, zinc, hydrogen arsenide, various acids and bases, noise, high temperature." (in 1st paragraph) and "Because the indium-exposed workers were typically the population occupationally exposed to indium compounds and toxic metals, ... (in 2nd paragraph)".

However, there is a note in the Manuscript (R2) on the selection criteria in the Methods section as "The selection criteria of the study population for this study (1) no history of occupational exposure to other metals, …". In other words, the selection criterion is that the study subjects have no exposure to other metals. This is not consistent with the description in the introduction or the rebuttal letter (R1). Please clarify your exposure to other metals.

*17-r2 How to select the study subjects

The method of selection of study subjects is not clearly described. In particular, the selection procedure for the unexposed workers remains unclear. Clarity on how study subjects are selected is essential to consider selection bias and generalizability. Lack of clarity prevents the reader from properly considering selection bias and generalizability. Please clearly describe the selection of the study subjects.

What I believe to be missing are listed below.

*17-r2-(1). How to select non-exposed persons

In a rebuttal letter (R1), the authors wrote, "The control subjects were office workers from another factory who were note exposed to occupational hazards. They were volunteers, and were matched with the exposed workers for age, sex and smoking habits".

In response, I wrote in Rev-com(R1) comment No *17-r1, "Please explain this information in the body of the manuscript as well as in the rebuttal letter. You mentioned that you matched workers at the indium plant (exposed group) and controls (non-exposed group) with age, gender and smoking history, but the And, there is a difference in the percentage of smokers between the groups , even though they were matched. please add an explanation for this in the main text."

In a rebuttal letter (R2), the authors wrote, "Information on the control subjects has

been supplemented in the body of the manuscript." The author had Added an explanation about the selection criteria of the study population and added an explanation for the difference in the percentage of smokers between the groups."

The authors have added selection criteria in manuscript (R2), but these are for the study subjects (= Indium-exposed workers + unexposed workers) rather than for the "unexposed workers (= Control subjects)" selection criteria. As a result, the manuscript(R2) does not describe the selection of unexposed workers in detail.

First, it does not state that they are volunteers as the authors wrote in rebuttal letter (R1). If the study subjects are volunteers, rather than randomly selected or selected by the researcher, this should be stated in the main text.

Secondly, the manuscript(R2) does not mention matching of unexposed workers. If the unexposed workers were selected by matching, then the matching methods and conditions should be included in the main text.

Also, please respond to the clarification noted in Rev-com(R1) comment No *17-r1. Please also refer to the commentary in STROBE 13. Participants.

- Vandenvbroucke JP et al. Strengthening the Reporting of Observational Studies in Epidemiology (STROBE):Explanation and Elaboration. (2007). Epidemiology. 18:805-835.

*17-r2-(2). Number of excluded subjects.

In the manuscript (R2), "the selection criteria of the study population for this study" was added, including "(3) complete information of outcome parameters (both in serum and urine)" and "(4) excluding outliers of each parameter". Please describe the number of subjects excluded because of criteria (3) and (4).

*17-r2-(3). Definition of Outlier

Please explain the definition of "outliers" in "(4) excluding outliers of each parameter".

*17-r2-(4). Need for flow diagram

Considering the above comments 17-r2-(1), 17-r2-(2), and 17-r2-(3), there were 120

study subjects for statistical analysis, but I think it is difficult to describe how they were selected and/or excluded in text alone. Therefore, I think a flow diagram is necessary.

See also the explanation in STROBE 13(c) Consider the Use of a Flow Diagram.

- Vandenvbroucke JP et al. Strengthening the Reporting of Observational Studies in Epidemiology (STROBE):Explanation and Elaboration. (2007). Epidemiology. 18:805-835.

17-r2-(5). Selection Bias and Generalizability

Clarity on how subjects are selected is important to consider selection bias and generalizability. Manuscript(R2) does not present enough information to consider these. The authors should clearly state how you selected the subjects and add a note about selection bias and generalizability in the Discussion section.

Please indicate the magnitude and direction of the impact of the bias caused by the selection of the study subjects on the results (selection bias) and describe the relative importance of the bias compared to other biases.

Please also indicate the extent to which the results obtained in this study can be adapted to other populations as generalizability.

See also STROBE 19 Limitations and 21 Generalizability Commentary.

- Vandenvbroucke JP et al. Strengthening the Reporting of Observational Studies in Epidemiology (STROBE):Explanation and Elaboration. (2007). Epidemiology. 18:805-835.

[Concerns of the newly additional parts]

37. about Table 2.

Controls subjects are not exposed, so the years of exposure should be zero (in the table, the average is 8.97)." Isn't "years of exposure (years)" the "employment period (years)"? Please check it.

38. about Table 5.

The authors found that "With regard to the degree ... Se (115%) and Zn (30%). (2nd paragraph of Correlation analysis of elements in serum or urine)". In addition to indium, there are references here to ten other elements (Al, Be, V, Cd, Cr, Mn, Mg, Mo, Se, and Zn). And the authors use this description as a reason to further analyze the correlation of the 15 elements. " Therefore, we furthre analyzed the correlation between the changes of the 15 elements ... (Table 5). (2nd paragraph of Correlation analysis of elements in serum or urine)".
Although the reason is stated for 10 elements, Table 5 that 15 elements are listed in the correlation is not logical (As, Cs, Li, Tl, and Rb are added without explanation). A logical explanation is required to explain why the 15 elements were selected from the 27 elements.

39. position of the Limitation

Limitation was listed in the Conclusion section in manuscript (R2). Limitation is not the conclusion of this study and should be listed in the Discussion section.

40. the Terms used in the selection criteria.

In criteria (3) and (4), the word "parameter" is used. Please consider whether this word is appropriate. The word "parameter" does not mean "measured value".
