# Supplementary material for: Biomonitorization of concentrations of 28 elements in serum and urine among workers exposed to indium compounds
Source: PLoS One. 2021 Feb 22;16(2):e0246943. doi: 10.1371/journal.pone.0246943 (PMC7899351; doi:10.1371/journal.pone.0246943)
Supplement: Attachment — Submitted filename: Response to Reviewers.docx [file pone.0246943.s003.docx]

**Reply to revision**

**Responses to Editor:**

Thank you for the opportunity to revise my paper. An explanation of the additional requirements below:

1. I read the PLOS ONE style templates carefully, and ensure that my manuscript meets PLOS ONE's style requirements.
2. Thank you very much for pointing out some problems. In my revision, I cite all my sources (including my own works), and make certain appropriate readjustments to some texts. But the author still wants to explain the difference between our paper and the following previous publications.

Publication 1：Title: Alteration of saliva and serum concentrations of manganese, copper, zinc, cadmium and lead among career welders. The differences between our paper and the publication are research objects (indium-exposed workers are different from career welders), biological samples (serum and urine rather than saliva), and analysis elements (28 elements are far more than 5 elements).

Publication 2：Title: Occupational Exposure to Welding Fume among Welders: Alterations of Manganese, Iron, Zinc, Copper, and Lead in Body Fluids and the Oxidative Stress Status. The differences between our paper and the publication are research objects (indium-exposed workers are different from welders), the treatment method of biological samples (the digestion solution direct dilution method is simpler and more accurate than microwave digestion), and analysis elements (28 elements are far more than 5 elements).

Publication 3：Title: Trace elements and carcinogenicity: a subject in review. The differences between our paper and the publication are article type (research article is completely different from review article in essence), main idea of the article (one is to study the detection and analysis of 28 elements in indium-exposed workers, the other is to study the relationship between trace elements and carcinogenicity).

Publication 4：Title: Inhibition of the WNT/β-catenin pathway by fine particulate matter in haze: Roles of metals and polycyclic aromatic hydrocarbons. The author has never read this publication. Moreover, through the abstract of that article, I found that the research content of that article is metal and polycyclic aromatic hydrocarbons in haze. The detection objects of our paper are indium-exposed workers, which is essentially different from that article.

3. I have attached a questionnaire as part of this study. I upload this as a separate file labeled 'Questionnaire'.
4. The corresponding author has an ORCID iD and has completed the Authorization process and have been logged in to Editorial Manager.

**Response to Reviewers:**

**Response Reviewer #1:**

Abstract:
When listing the elements in the results, the author has carefully revised the specific samples of each element, such as serum, urine or both.

Methods:

Demographics have been changed to the Results section.
In our study, individuals who had consumed alcohol more than twice a week during the previous 6 months were defined as drinkers.

Discussion:

In order to control the workers' serum indium level, we should control the environmental contamination from indium facilities and do a good job of workers' personal protection. Therefore, the author address solutions at the end of the first paragraph of the discussion.

Our results show that, some elements/metals were higher in one specimen (serum or urine) but lower in the other in indium-exposed workers, this increase or decrease could partly be attributed to the possible action of indium on trace elements metabolism. Perhaps the higher exposure in the indium exposed workers will really make this group of workers more vulnerable to metal toxicities.

‘At present, it is uncertain whether the concentration of indium in serum or urine can be used as a biological indicator or biomarkers of indium exposure’: this statement is not correct, the author has corrected this sentence in the discussion section.

**Response Reviewer #2:**

1. The author has made some modifications to some paragraphs in the Introduction section and the Discussion section.
2. By carefully reviewing previous studies, the author increases the number of cited references.
3. I'm very sorry, I didn't find the error you said. Please point it out.
4. The author has made some concise modifications to the discussion part.
5. The author submitted a Research Checklist.
6. Thank you for the opportunity to check my English grammar.
7. This study was aimed to assess inddium and other 27 elements in serum and urine. The total is 28 elements.
8. The author has added the method of the ambient indium level measure in the Abstract-Method section.
9. "the variation in ... among study populations.": this conclusion is inaccurate and has been deleted.
10. "Linear regression analysis": the "r" was a standardized regression coefficient. The standardized regression coefficient refers to the regression coefficient calculated after the data is standardized (minus the mean value and dividing the variance). It can eliminate the influence of dimension and order of magnitude after the standardized data, so that different variables are comparable. Therefore, the standardized regression coefficient can be used to compare the effect of different independent variables on dependent variables.
11. "we have validated ... (ICP-MS)." : in this part of the content, the author's expression may be wrong, there is no “validated results” here. The author has made corrections in the paper.
12. "To the best ... indium-exposed population.": this part has been changed to the Discussion section.
13. "toxic metals": the indium-exposed workers were exposed to other toxic metal, too. It can be seen from the first paragraph of the Introduction section, “In the process of indium smelting, there are occupational hazards such as dust, lead, arsenic, cadmium, indium, zinc, hydrogen arsenide, various acids and bases, noise, high temperature.”.
14. "Hence this study ... its compounds exposure.": This sentence is repeated with the Discussion section and has been deleted.
15. Demography has been placed in the Results section.
16. During operation, the exposed workers wear dust-free clothes, while maintenance workers wear full face filter cotton, goggles and protective gloves.
17. The control subjects were office workers from another factory who were not exposed to occupational hazards. They were volunteers, and were matched with the exposed workers for age, sex, and smoking habits.
18. General characteristics of the 120 study populations in the control and indium exposure group are summarized in Table 1. The distribution of age and frequencies of sex, smoking and drinking were not significantly different among the two groups.

Table 1	General characteristics and lifestyle habits in study populations.

| Variable | Controls | Wokers | statistics | *P* |
| --- | --- | --- | --- | --- |
| Number | 63 | 57 |  |  |
| Age (year,$\bar{x}$±s) | 39.32 ± 11.37 | 37.82 ± 9.34 | 0.77 | 0.44^a^ |
| Sex (male/female,%) | 46/17(73) | 41/16(72) | 0.02 | 0.89^b^ |
| Smoking (yes/no, %) | 31/32(49) | 21/36(37) | 1.86 | 0.17^c^ |
| Drinking (yes/no, %) | 20/43(32) | 18/39(32) | 0.00 | 0.98^c^ |
| Years of exposure (year,$\bar{x}$±s) | 8.97 ± 8.39 | 8.31 ± 7.54 | 0.45 | 0.65^a^ |

^a^ Ln-transformed for statistical inference. ^b^ One-way ANOVA. ^c^ χ^2^ test.

19. The author has merged the overlap together.
20. The author has added the approval number.
21. Yes, it is 15%, the author has corrected the mistake.
22. The “local clinics” refers to the local occupational disease prevention and control hospital where the indium factory is located.
23. The author has corrected the incorrect citation style.
24. The author has inserted a space between "℃" and "u".
25. The author has put the information of 28 elements in Table 1.
26. The author has used Welch's t test to analyze the data.
27. The author has changed the description of the error.
28. The differences between two means were analyzed by independent samples T test.
29. A multiple regression analysis has listed in Method section,and stated it.
30. The author has rewrited the paragraph to brief description of the airborne indium level.

**Response Reviewer #3:**

1. The author has explained in the second paragraph of the Discussion section why serum and urine samples were selected for in vivo biomonitoring of indium and its compounds.
2. The author has attached a paragraph about the quality assurance of our results in the Method section.
3. The author has changed the unit of the airborne indium level into the same units, such as mg / m^3^.
4. As requested, the author has elaborated on this statement in the fifth paragraph of the Discussion section.
5. Through these research results, the author couldnot conclude that indium’s residence time in vivo is quite long that cause indium more hazard to human health. The author can only prove that the longer the working years of workers, the longer the exposure time of indium, resulting in the higher content of indium in serum or urine, which may cause more hazard to human health; however, this does not mean that the long accumulation time of indium in the body causes harm to human body.
6. I had readjusted my English grammar. If you feel there are any problems, please let me know as soon as possible, and I will try my best to adjust it.
