# Supplementary material for: Biomonitorization of concentrations of 28 elements in serum and urine among workers exposed to indium compounds
Source: PLoS One. 2021 Feb 22;16(2):e0246943. doi: 10.1371/journal.pone.0246943 (PMC7899351; doi:10.1371/journal.pone.0246943)
Supplement: Attachment — Submitted filename: Response to Reviewers.docx [file pone.0246943.s004.docx]

**Reply to revision**

**Responses to Academic Editor:**

Thank you for the opportunity to revise my paper. I am honored to have the opportunity to talk about the indium levels measured by the subjects in the control group. Through consulting the previous literature, the author found that the geometric mean of indium in blood of general population was lower than the detection limit. Therefore, when the present results appeared, the author thought that there was something wrong with the detection. After careful analysis and verification, there is no problem with our detection method, and the selected control population has no previous occupational exposure. As a result, we analyzed that the possible reason was background indium exposure. The population in our research is in Liuzhou City, Guangxi Province, China, which is the richest and most abundant indium resources in China. They are mainly distributed along the river. Although there was no occupational indium exposure in the control population, the indium pollution in river or soil was not ruled out. If possible, we would like to study the serum or urine indium levels of local non occupational indium-exposed populations.

DOI：[dx.doi.org/10.17504/protocols.io.bne2mbge](https://dx.doi.org/10.17504/protocols.io.bne2mbge)

**Response to Reviewers:**

**Response Reviewer #1:**

why the control subjects would have measurable indium in blood?

Thank you for the opportunity to talk about the indium levels measured by the subjects in the control subjects. Indium is not a trace element and the author found that the geometric mean of indium in blood of general population was lower than the detection limit by consulting the literature. But at the same time, there have not been large studies to measure blood indium in unexposed populations. Therefore, up to now, there are very few reports on the level of indium in normal human serum or blood. First of all, the author can confirm that the detection method in this paper is appropriate and the selected control population has no previous occupational exposure. As a result, we analyzed that the possible reason was background indium exposure. The population in our research is in Liuzhou City, Guangxi Province, China, which is the richest and most abundant indium resources in China. Guangxi has become the largest indium production base in China due to its advantaged indium resources, and is known as "indium capital" in the world. Those indium bearing mine are mainly distributed along the river and there are many local production and processing enterprises. Although there was no occupational indium exposure in the control population, the indium pollution in river or soil was not ruled out. Of course, this is only our speculation. If possible, we would like to study the serum or urine indium levels of local non-occupational indium-exposed populations.

**Response Reviewer #2:**

Thank you for the opportunity to revise my paper. An explanation of the points is below:

1. *3-r1. multiplicity of statistical tests
   Thank you very much for your detailed description of the statistical problems in our study. The inevitable limitation of our study is that the sample size is too small. In order to distinguish between cases where there is a truly significant difference and cases where the difference appears to be significant by type I errors, the author reduced the number of calculated p-values. Therefore, the author makes some modifications in the analysis of the Results section (Table 5).
2. *5-r1. Check List
   Thank you for sending me a link to the STROBE checklist. The author has carefully read each checklist item of the STROBE checklist to use with this article.
3. *10-r1. standardized regression coefficient

The author has added and explained this description in the Method section.

4. *16-r1. personal protective equipment

The author has explained this in the Method section (Subject).

5. *17-r1. Control subject

Information on the control subjects has been supplemented in the body of the manuscript.

The author has added an explanation about the selection criteria of the study population and added an explanation for the difference in the percentage of smokers between the groups.

6. *18-r1 Comparison between workers and control

The author has added this Table for the manuscript.

Table 2	General characteristics and lifestyle habits in study populations.

|  |  | Controls (n=63) | Workers (n=57) |
| --- | --- | --- | --- |
| Age (years) | Mean ± SD | 39.32 ± 11.37 | 37.82 ± 9.34 |
| Sex | Male | 46 (73.0) | 41 (71.9) |
|  | Female | 17 (27.0) | 16 (28.1) |
| Smoking | Non-smoker | 32 (50.8) | 36 (63.2) |
|  | Smoker | 31 (49.2) | 21 (36.8) |
| Drinking | Non-drinker | 43 (68.3) | 39 (68.4) |
|  | Drinker | 20 (31.7) | 18 (31.6) |
| Years of exposure (years) | Mean ± SD | 8.97 ± 8.39 | 8.31 ± 7.54 |

[New comments]

7. Limitation

The author has added the limitations of this study in the Abstract and Discussion section.

8. Duplicate expressions.

The author has merged the duplicate expressions.

9. emotional expression

The author has changed the way of expression.

10. Description to be included in the Result section

The author has put the description of results in the Results section

11. Conclusion section

The author has deleted “broad”.

12. Typo and unformal word

I'm very sorry. This is the author's mistake. The author has corrected it.
