# Supplementary material for: Biomonitorization of concentrations of 28 elements in serum and urine among workers exposed to indium compounds
Source: PLoS One. 2021 Feb 22;16(2):e0246943. doi: 10.1371/journal.pone.0246943 (PMC7899351; doi:10.1371/journal.pone.0246943)
Supplement: Attachment — Submitted filename: Response to Reviewers.doc [file pone.0246943.s006.doc]

**Reply to revision**

**Responses to Academic Editor:**

Thank you for the opportunity to revise my paper.

**Response to Reviewers:**

**Response Reviewer #2:**

Thank you for the third opportunity to revise my paper. An explanation of the points is below:

[Ongoing concerns]

*3-r2. Multiple statistical testing Problem (False positive rate will be extremely high)

Thank you very much for your careful explanation and explanation of this problem, so that the author has the opportunity to learn a lot of analysis methods.

The author carefully interpreted the p value, added that “this is an exploratory study” in the Method section, added it as a Limitation in the Discussion section.

*13-r2. Are the study subjects exposed to any other toxic metals?

The selection criteria for the study population in this study were indeed no history of occupational exposure to other metals. The author’s response stated "the indium-exposed workers were exposed to other toxic metals, too." , which means that lead, arsenic, cadmium and other toxic metals exist in the process of indium smelting, workers may also be exposed to lead, arsenic, cadmium and other toxic metals when they are exposed to indium. However, this does not mean that the indium-exposed workers we chose are also occupationally exposed to other toxic metals.

*17-r2 How to select the study subjects

Please see the flow diagram.

*17-r2-(1). How to select non-exposed persons

I'm sorry, maybe there is something wrong with my language expression in my rebuttal letter (R1). The study subjects (= Indium-exposed workers + unexposed workers) are not volunteers. The Indium-exposed workers were selected from an indium ingot production plant (Guangxi, China) who were mainly exposed to indium metal. The unexposed workers (Control subjects) were a random sample of all volunteers derived from another nearby factory, who were office workers and had no history of occupational exposure to indium and other metals. The selected control subjects were matched to the age, sex distribution, average employment history, smoking status and drinking habits with indium-exposed workers.

Respond to the clarification noted in Rev-com(R1) comment No *17-r1: The study subjects were initially matched by inclusion criteria, the number of person is 69:69; but after a series of exclusion criteria, there were some differences between indium-exposed workers and control subjects (the number of person is 57:63).

*17-r2-(2). Number of excluded subjects.

The author described the number of subjects excluded because of criteria (3) and (4).

*17-r2-(3). Definition of Outlier

In our analysis of serum elements of all study subjects, we found that, the serum magnesium level of one control worker was abnormally low (3.8 mg/L). But the worker did not know about his problem. After our discovery, he went to the hospital for further examination and found that he had hypomagnesemia. Thus observations in this worker who had outliers outcome were excluded.

*17-r2-(4). Need for flow diagram

The author saw the explanation in STROBE 13(c) Consider the Use of a Flow Diagram; and supplemented a flow diagram.

Figure 1. Flow of Indium-exposed workers and unexposed workers in the study.

*17-r2-(5). Selection Bias and Generalizability

The author added “Potential confounders and sources of bias” in the Method section, added “Limitations and Generalization of the study results” in the Discussion section.

[Concerns of the newly additional parts]

37. about Table 2.

What the author wants to express is "employment period (years)". Changes have been made in Table 2.

38. about Table 5.

After checking and verifying Table 3, the author found some errors and corrected them in Table 5.

39. position of the Limitation

The author has listed the limitations in the Discussion section.

40. the Terms used in the selection criteria.

The author has changed the word "parameter" to "measured value".
