# Supplementary material for: Biomonitorization of concentrations of 28 elements in serum and urine among workers exposed to indium compounds
Source: PLoS One. 2021 Feb 22;16(2):e0246943. doi: 10.1371/journal.pone.0246943 (PMC7899351; doi:10.1371/journal.pone.0246943)
Supplement: Attachment — Submitted filename: Response to Reviewers.doc [file pone.0246943.s007.doc]

**Reply to revision**

**Responses to Academic Editor:**

Thank you for the opportunity to revise my paper.

**Response to Reviewers:**

**Response Reviewer #2:**

Thank you very much for your patient guidance and careful revision of this paper, so that the author has the opportunity to learn a lot of writing and analysis methods. An explanation of the points is below:

[Minor concerns]
41. The English word "volunteer" should be avoided.

The author has changed the word "volunteer" to "candidates".

42. Elemental symbols or English words.
The author has used the elemental symbols instead of the English words.
